# Young people’s perceptions of youth-oriented health services in urban Soweto, South Africa: a qualitative investigation

**DOI:** 10.1186/s12913-014-0625-y

**Published:** 2014-12-05

**Authors:** Brittany Schriver, Kathryn Meagley, Shane Norris, Rebecca Geary, Aryeh D Stein

**Affiliations:** Department of Global Health, Rollins School of Public Health, Emory University, Atlanta, GA USA; Department of Behavioral Sciences and Health Education, Rollins School of Public Health, Emory University, Atlanta, GA USA; MRC/Wits Developmental Pathways for Health Research Unit, Department of Paediatrics, University of the Witwatersrand, Johannesburg, South Africa; Department of Population Studies, Faculty of Epidemiology and Population Health, London School of Hygiene and Tropical Medicine, London, England UK

## Abstract

**Background:**

In 2006, the South African Department of Health adopted and scaled-up loveLife’s Youth Friendly Services (YFS) initiative to a national policy to improve youth utilization of health programmes by strengthening community sensitisation and counselling services. As these services roll-out, alternative services to target young people are also becoming more popular. Success of any of these services, however, is dependent upon young people’s perceptions of these health services as a whole.

This paper aims to examine the knowledge and perceptions of current health services oriented towards young people and examine potential alternative approaches to health service delivery.

**Methods:**

The study was conducted in urban Soweto, South Africa. Twenty-five in-depth interviews were conducted between May-July 2012. Twenty-three of these were analysed according to modified grounded theory.

**Results:**

Knowledge of YFS was very low with no thorough knowledge of the programme’s purpose or activities. In general, young people were dissatisfied with the current health services in Soweto citing a lack of resources, long waiting times, and poor quality of care heightened by an underlying lack of choice and perceived inequity. When compared to alternative models of service delivery, no particular model was preferred over another.

**Conclusions:**

Greater knowledge of whether and to what extent local clinics in Soweto are implementing YFS standards is needed. If implemented, improved outreach and advertisement is suggested. In-service training of nurses should be prioritized with a focus on sensitivity and equitable treatment to all.

## Background

At 1.8 billion, there are now more young people (aged 10–24 years) than at any point in human history [1]. The adolescent health burden is particularly heavy in low and middle income countries, where young people comprise a greater proportion of the population than in high-income countries [2]. Nearly one-third of the population of South Africa falls between the ages of 10 and 24 years [3]. While young people are generally regarded as healthy, this is a period whereby individuals pass through physical, psychological, and sexual maturation that influence health and determinants of health later in life.

In South Africa, the average age at first intercourse is 15 years for boys and 14 for years for girls [4]. Nearly half of young women have given birth before the age of 20 with 66% reporting that the pregnancy was unwanted [5]. While HIV has affected all age groups, young people have been hit hardest, as HIV prevalence is now at 13.6% among young women and 4.5% among young men [6]. In spite of this, many young people do not use health services and have reported barriers when they do attend clinics [7]. To reduce morbidity and mortality related to young peoples’ vulnerability and resistance to seek care, greater effort needs to be made to address the unique attributes, needs, and priorities of this population.

In 1999, loveLife was established as a joint initiative of leading South African Non-Government Organizations (NGOs), private partnerships, and the South African government as a multi-faceted approach to a national HIV prevention and sexual health education programme [8]. This programme used a range of strategies including print and media education campaigns, peer education (groundBREAKERS), outreach and mobilisation, and 18 youth centres (Y-Centres) [4]. By 2001, the programme had expanded to all nine provinces and by 2005 was available in 305 health facilities throughout the country as the National Adolescent Friendly Clinic Initiative (NAFCI) [4]. The programme was scaled up to a national policy, adopted by the South African Department of Health in 2006 and renamed Youth Friendly Services [9]. This scale-up has proven difficult, in part due to the decentralised system in which it exists as well as the deeper history of inequities in services that continue to affect perceptions of quality and trust in the health system [10].

This paper aims to examine young people’s knowledge and perceptions of current health services oriented towards this age group, and to examine potential alternative models for health service delivery.

## Methods

### Setting and sampling

We conducted this study in Soweto, South Africa, an urban township situated to the southwest of Johannesburg. We purposively sampled twenty-three Black African young men and women from a subset of fifty BT20 cohort members. This subset had been randomly-selected from the full cohort to participate in a pilot of the periodic Young Adolescent Health Survey (YAHS). We selected the sub-set of participants for this study based on gender (14 female/9 male) and utilization of health services within the last six months (15 users/8 non-users). The BT20 cohort is the largest and longest running study of child and adolescent development on the African continent. Full details of the design of the cohort study are described elsewhere [11,12].

We used modified grounded theory to guide our work (as described by Borgatti [13] and Strauss and Corbin [14]). We conceptionalised theories through literature reviews that informed our data collection tools, while allowing additional themes to emerge throughout data collection and analysis.

### In-depth interviews

We developed an in-depth interview guide prior to the study that covered individual’s definitions of health, sources of health information and perceptions of current and potential health services. The guide was informed by a thorough review of the literature and refined through pilot interviews with local research staff.

We conducted five pilot interviews with members of a subset of the BT20 cohort to determine and refine the interview method. We piloted three optional interview methods: (a) semi-structured 1-on-1 interview between one of the Principal Investigators (PIs) and the participant in English, (b) semi-structured 1-on-1 interview between a Zulu-speaking research assistant and the participant in Zulu and English, and (c) interview between one of the PIs and the participant with the Zulu-speaking research assistant present for translation if participant felt more comfortable expressing themselves in Zulu. Pilot interviews indicated that participants were most comfortable expressing their opinions in option a.

Project staff contacted participants by phone and invited them to be interviewed. We obtained informed consent from all participants and held interviews in private interview rooms at the BT20 cohort offices. A range of topics were discussed including sources of health information, perceptions of current health services, and opinions on alternative health services. Daily meetings between the PIs assisted in the reflexive process during both the pilot and data collection phases.

We submitted protocol granted exemption by Emory University IRB (ID 58317). We were granted local ethics approval by the University of Witwatersrand under the BT20 approval (ID M120138).

### Data analysis

We conducted all interviews in English; interviews varied in length between 45 minutes and two hours. We recorded and transcribed all interviews verbatim. The two PIs independently read all transcripts and developed codebooks based on emerging themes. As guided by modified grounded theory, we developed codes deductively, informed by the themes of availability, accessibility, and acceptability as previously discussed in literature [7,15-17], and inductively from the iterative process of collecting and analysing interview data. Codebooks were synthesised and consolidated, and code definitions were agreed upon by both PIs. Inter-coder agreement was checked for three interviews; discrepancies in coding were discussed and a consensus was reached. The PIs coded and re-coded the remaining transcripts so that final transcript used for analysis included both of the PIs’ independently coded text. Data were coded and analysed using MAXQDA10 Qualitative Analysis Software.

### Limitations

This study utilized the BT20 cohort, which has been studied regularly for over 20 years, exposing participants to more regular screenings and health education than the general population of young people. This may have impacted answers related to knowledge of healthy behaviors and expectations of health services. Additionally, all participants were aged 21 or 22 at the time of the interview. While this falls in the age range of “young people” (aged 10–24), it falls in the upper limit and might not be representative of younger persons perceptions. Lastly, the data in this study may be subject to contextual effects that may affect data quality. This may include characteristics of the interviewer, differences in reactions to each PI interviewing, level of comfort with the interview process, and setting. Efforts were made to minimize these effects, through rapport building, the informed consent process, and reflexive meetings between the PIs after each interview.

## Results

We conducted a total of 25 interviews (including pilot interviews), 23 (14 female, 9 male) of which were used for data analysis. We did not use two pilot interviews (methods b&c) in analysis as they did not reflect the overall data collection methodology used (method a). Interviews captured perceptions and opinions for both recent users and non-users (reported visited within the past six months) of local health services (15 users/8 non-users), including knowledge and perception of YFS initiatives. Additionally, participant’s opinions on two potential alternative health service delivery methods were collected.

### Perception of current health services

Young people were generally dissatisfied with the current public health services in Soweto. Dissatisfaction was linked to a lack of resources, long waiting times, and poor quality of care. These reflect well-documented barriers to acceptable health care services [7,15]. Staffing shortages, insufficient diagnostic equipment (such as x-rays) and drug stock-outs were reported. Because of frequent drug stock-outs, clinics often only offered basic medications such as antibiotics or generic painkillers like Panado© which are readily available at small shops and supermarkets. Participants felt that going to a clinic was an unnecessary step that did not always result in better outcomes.

*‘They’ll give you Panado©. Any pain killer or they’ll just say they’re out of stock. So you might as well just buy your own pain killer and not go to the clinic’ (Male).*

Youth who did visit local clinics often faced extensive waiting times. Young people interviewed reported waiting between 30 minutes and several hours to see a nurse or doctor. Discontent was aggravated by a perception that their wait was often a result of nurses taking prolonged tea-breaks, leaving early, or dismissing their duties. Overall, participants felt nurses were rude, did not establish a sense of confidentiality or show respect to their needs. Some differentiated this behaviour from doctors, indicating that while doctors were scarce they perceived them to be more attentive and committed to their jobs.

Dissatisfaction was heightened by underlying feelings of inequity in choice and access to quality services. Participants described a distinct variability and hierarchy of services. Private clinics were viewed as the pinnacle of health care services, described as trustworthy, clean, fast, reliable, and better staffed and stocked than publicly funded healthcare facilities. Those who had accessed private health services (6 interviewees) indicated not only satisfaction with the services, but gratefulness in their ability to pay and avoid the public system. Chemists or pharmacists, who can do quick diagnosis and prescribe over-the-counter drugs, were considered to be better than public health facilities but not as good as private clinics. When neither of these services was accessible due to financial barriers, young people relied on free public services.

When faced with attending a public clinic, participants discussed three action strategies: avoidance, fatalistic acceptance, and manipulation of the system. When not feeling well, patients first reported avoiding the public clinics. Those interviewed primarily relied upon home remedies or information from libraries, the Internet and online chat rooms. Utilisation of the public health system often only came after all other treatments failed and access to private services was financially infeasible. At this point, there was a sense of fatalistic acceptance.

Interviewer: *‘At what point would you think about going to the clinic?’*

Participant*: ‘When it is serious. But I don’t like clinics so I don’t usually go.’*

Interviewer*: ‘What makes it serious?’*

Participant*: ‘When you have taken all other methods and they don’t help… you must go’ (Female).*

*“Ah, it’s it’s it’s quite bad. Some people don’t even have the money to go to private doctors, so they have no choice. They they have to go there, and be in the queue for long hours. It’s not it’s not good” (Female).*

Some participants discussed being gossiped about, judged, or even turned away if they were perceived to have the ability to afford private services. They were told they were trying to ‘*take advantage*’ of free services that are not ‘*meant for them*’. Others discussed feeling that if clinic workers judged them to be of a lower class, they treated them as if they were deserving of poor services and treatment. When faced with this type of treatment, young people felt they did not have the social capital to make complaints or demand better service because the services were free. One patient discussed how this perpetuates poor treatment from the nursing staff because they will not be held accountable for their behaviour.

*“Cause some—they do go to the clinic but then, like I said, the other problem is that you find people who are judgmental or rude. You know? So that’s the reason why they are afraid to go” (Female).*

*‘The one in [neighbourhood 1], like, there are nurses who are treating white people, colours, like there are all types of people who are going there. So I think maybe first that the advantages is that you have people who treat people good because they know there are other people who will generate results. When they are treated bad, the report it right away. So I feel like they should always treat us like people. [At neighbourhood 2], they take advantage. There are only black people there. They take advantage of that… they just mistreat people because they are at the local’ (Female).*

There were, however, clinics that participants reported as being ‘good’ clinics, those that were regarded as having greater resources, shorter waiting times, and friendlier staff. Access to these clinics was limited. A National Department of Health policy states that patients must visit their nearest clinic first and get subsequent referrals if the patient would like to go elsewhere [18]. To access ‘good’ clinics, several participants discussed manipulating the system by using local addresses or another family member’s information.

*“Yeah so you just go there and just write my name and the new address where I’ll stay at that moment in that village. So just write there and they’ll say ‘ok, fine”(Male).*

Participant*: ‘Well… there are some good clinics…but these days they’re strict at clinics. They’ll check your address so you can’t go to any clinic you want. Cause then you want to go to the favourite clinic, so they get packed there and other clinics are empty. So now they first check your address before attending you’.*

Interviewer*: ‘How do you feel about that?’*

Participant*: ‘No… it’s bad. Because we go there knowing we get the better service than the other clinics. But we can’t go… unless I change my address’ (Female).*

### Knowledge of YFS initiatives

Overall, knowledge of YFS programmes was very low; of 23 participants interviewed only three reported ever hearing about ‘youth-friendly services,’ none of whom were able to express extensive knowledge of the programme’s purpose or activities. After the interviewer described the YFS programme, some participants acknowledged these services might exist but recognised the lack of knowledge as a problem.

*‘I think there is one at our clinic, but I’ve never been to that… I’ve never heard people say, speaking anything about or going there. Yeah, like cause sometimes they don’t know about it. It’s just there’s a centre there, people don’t really know what’s going on… they’re not really clued in on what’s going on’ (Female).*

*“I think the services are ok…the only thing is: are youth attending them? You know, going and learning about them? The youngsters, they need to be more, um, what can I say, they need to be made aware of that, that these services are there for us to go” (Female).*

Most participants agreed that it sounds like a good programme, and that nurses should be trained on respect for all persons, confidentiality, dedication, as well as proper sensitivities to young people’s needs. For the most part these needs were related to sexual health, including sexually transmitted infections, HIV/AIDS, and pregnancy. Additionally, general health education, diabetes, and counselling were brought up as important health issues facing these young people.

YFS was developed from the loveLife programme, so participants were also asked if they had ever heard of the loveLife’s peer educators, groundBREAKERS. Eighteen of the twenty-three participants indicated that they had heard of groundBREAKERS, the majority of whom (14 of the 18) correctly matched a description to the programme. Overall, participants perceived the groundBREAKERS programme positively, and found them relatable, trustworthy and a good source of health education.

### Attitudes toward alternative health services

This study also examined the participant’s opinions of two alternative health service delivery systems: School Based Health Clinics (SBHC) and Community Health Workers (CHW). There was no particular service that was preferred. While most of the participants were excited to discuss these potential services, a few participants seemed sceptical about the likelihood of these services being implemented in Soweto - potentially reflecting their feeling of disenfranchisement with current health service interventions.

*‘No. It could work. I’m just thinking training nurses to be friendly and non-judgmental- Oh! [raises eyebrows in disapproval]…’ (Female).*

In general, reactions to SBHC were positive; all but one young person interviewed said they would have liked to have had a SBHC at their school when they were younger. Perceived benefits of a SBHC included fewer missed class hours to visit clinics, improved educational performance, and greater health awareness. While there was no consensus regarding the demographic characteristics (age, gender) of potential SBHC staff, participants generally agreed that independent nurses not affiliated with the school should staff SBHC, pointing to uneasiness with the idea of teachers having access to the health information of the students. Another concern that arose was related to a sense of inequity- that if these services were in schools they would then only be available to current students and not be available to out-of-school youth or young adults that are no longer school-aged. Some also discussed concern for older people being excluded from these services, and suggested that more outreach services should be available to them.

In general, the CHW model most accommodated the feeling for greater equity in access to health services. Participants liked the idea of having health services visit them in their home environment, however it was widely agreed that CHWs should not target certain houses or skip houses, to avoid stigma, promote confidentiality and ensure no one needing health services was missed.

*‘For example, they go to this house and maybe from this house you’re skipping this one and going to that one [points to three imaginary houses]. What if this person would die today? [pointing to skipped house] If I did come it would be fine and I would be seeing he would be having sickness’ (Male).*

Health education and HIV testing and treatment were among the services participants would most like to see offered by CHWs. Other services that were discussed included general check-ups, health education and assessments for obesity, blood pressure, diabetes, and illness. While there was little consensus regarding the demographic characteristics (age, gender) of potential CHWs, participants agreed that CHWs should be clearly identifiable (with a uniform or badge) so that safety could be ensured.

## Discussion

### Perceptions of current health services

Participants were generally dissatisfied with local public clinics, citing reasons reflecting literature, including the lack of availability, accessibility, and acceptability [7,15-17,19]. Negative perceptions stemmed primarily from interactions with service staff and the availability of resources, rather than the condition or accessibility of facilities. Studies from other sub-Saharan African countries (Kenya, Burkina Faso, Ghana, Malawi, Uganda and Mozambique) suggest that by improving existing public health systems (via service and resource availability, provider attitude) youth report more positive attitudes towards those services and greater uptake [20-22]. These findings suggest there is an opportunity to provide high-quality youth-oriented health services via the existing system and that more resources should be invested in training staff and ensuring adequate stock supplies over renovating clinics.

There also seemed to be underlying feelings of inequity in choice and access to more acceptable services. Participants reported feeling that the services that were available to them were of lower quality because they were free and intended for those of low socio-economic status. A recent study by Harris, et al. suggests these negative perceptions of inequality could stem from policies established in the Apartheid era [23]. When apartheid ended, the national department of health reallocated resources from previously advantaged provinces to historically disadvantaged provinces [10]. However, limited capacity matched with low resources has led to frequent drug stock-outs, inadequate or no diagnostic equipment, and insufficient skilled staff in some local clinics [24]. These disparities, matched with policies that require patients to visit their nearest clinic [18] regardless of quality, may contribute to feelings of inequity.

### Knowledge of YFS initiatives

Among those interviewed, there was no thorough knowledge of the YFS programme. Lack of awareness of YFS has two underlying potential causes: (1) the clinics used by the participants do not implement or enforce YFS standards or (2) the clinics do adhere to YFS standards, but young people do not recognise them. The national DoH had aimed for 70% of primary healthcare facilities to be implementing the YFS program by 2012/13 [25], however a recent study in rural South Africa found only one of the eight publicly-funded primary healthcare facilities provided YFS [26]. This suggests greater implementation of YFS standards is needed with continued monitoring and evaluation of implementation. The study did find similar positive attitudes towards loveLife and the groundBREAKERS programme found in previous studies [19]. The YFS programme should consider utilising similar outreach and communications approaches to those used by loveLife.

### Attitudes towards alternative services

When presented with alternative health service delivery systems, young people displayed no overall preference for the YFS, SBHC or CHW models. Evidence supporting one delivery system over another is weak [27,28]. Further research should examine the cultural-appropriateness, logistic feasibility, and cost-benefit of each of these delivery systems before further scale-up of these programmes.

Reflective of the 2001 Erulkar study [19], participants did not express any preferences in relation to the demographic characteristics of service providers. The attitudes of healthcare workers were the most influential factors participants would like to see improved. These results suggest that recruiting people with certain characteristics to be health workers may be less important than improving the training and attitudes of existing health workers [19]. Furthermore, equity continued to be a driving theme among the discussion of alternative service delivery systems, indicating that staff trainings should emphasise equitable treatment.

## Conclusion

In conclusion, greater knowledge of whether and to what extent local clinics in Soweto are implementing YFS standards is needed. If clinics are implementing YFS, greater effort should be made to publicise the purpose and standards expected of YFS and ensure awareness where YFS clinics exist. This would have two purposes- to alert young people of clinics that might suit their needs better, and keep nurses and clinic staff accountable to the advertised standards. Lastly, training for health-service workers should emphasise respect and equitable treatment while ensuring that health facilities are adequately stocked with the whole range of health services.

## References

[1] Mathers C, Stevens G, Mascarenhas M (2009). Global health risks: mortality and burden of disease attributable to selected major risks.

[2] Sawyer SM, Afifi RA, Bearinger LH, Blakemore S-J, Dick B, Ezeh AC, Patton GC (2012). Adolescence: a foundation for future health. The Lancet.

[3] Statistics South Africa (2012). Census 2011 Statistical Release.

[4] Ashton J, Dickson K, Pleaner M (2007). The evolution of the national adolescent friendly clinic initiative in South Africa.

[5] Panday S, Makiwane M, Ranchod C, Letsoalo T (2009). Teenage Pregnancy in South Africa: With a Specific Focus on School-going Learners: Executive Summary. Child, Youth, Family and Social Development, Human Sciences Research Council.

[6] UNAIDS (2010). Report on the Global AIDS Epidemic.

[7] Richter MS, Mfolo V (2006). The perception of South African adolescents regarding primary health care services. Sci World J.

[8] Pettifor AE, MacPhail C, Bertozzi S, Rees HV (2007). Challenge of evaluating a national HIV prevention programme: the case of loveLife, South Africa. Sex Transm Infect.

[9] Department of Health Republic of South Africa (2012). National Adolescent and Youth Friendly Health Services Strategy 2012.

[10] Pillay Y (2001). The impact of South Africa’s new constitution on the organization of health services in the post-apartheid era. J Health Polit Policy Law.

[11] Richter L, Norris S, Pettifor J, Yach D, Cameron N (2007). Cohort profile: Mandela’s children: the 1990 birth to twenty study in South Africa. Int J Epidemiol.

[12] Richter LM, Norris SA, De Wet T (2004). Transition from birth to ten to birth to twenty: the South African cohort reaches 13 years of age. Paediatr Perinat Epidemiol.

[13] Borgatti S (2005). Introduction to grounded theory.

[14] Corbin JM, Strauss A (1990). Grounded theory research: procedures, canons, and evaluative criteria. Qual Sociol.

[15] Juszczak L, Melinkovich P, Kaplan D (2003). Use of health and mental health services by adolescents across multiple delivery sites. J Adolesc Health.

[16] Tylee A, Haller D, Graham T, Churchill R, Sanci L (2007). Youth-friendly primary-care services: how are we doing and what more needs to be done?. Lancet.

[17] Hock‐Long L, Herceg‐Baron R, Cassidy AM, Whittaker PG (2003). Access to adolescent reproductive health services: Financial and structural barriers to care. Perspect Sex Repro H.

[18] Doherty J, Rispel L, Webb N (1996). Developing a plan for primary health care facilities in Soweto, South Africa. Part II: Applying locational criteria. Health Policy Plan.

[19] Erulkar AS, Beksinska M, Cebekhulu Q: *An Assessment of Youth Centers in South Africa*. Population Council & Reproductive Health Research Unit; 2001. https://www.iywg.org/sites/iywg/files/SouthAfrica_Youth_Centers.pdf.

[20] Biddlecom AE, Munthali A, Singh S, Woog V (2007). Adolescents’ views of and preferences for sexual and reproductive health services in BurkinaFaso, Malawi and Uganda, Ghana. Afr J Reprod Health.

[21] Hainsworth G, Zihalo I (2010). From inception to large scale: the Geracao Biz programme in Mozambique.

[22] Godia PM, Olenja JM, Hofman JJ, van den Broek N (2014). Young people’s perception of sexual and reproductive health services in Kenya. BMC Health Serv Res.

[23] Harris B, Eyles J, Penn-Kekana L, Thomas L, Goudge J (2014). Adverse or acceptable: negotiating access to a post-apartheid health care contract. Global Health.

[24] Harris B, Goudge J, Ataguba J, McIntyre D, Nxumalo N, Jikwana S, Chersich M (2011). Inequities in access to health care in South Africa. J Public Health Pol.

[25] Department of Health Republic of South Africa (2010). National Department of Health Strategic Plan 2010/11–2012/13.

[26] Geary RS, Gómez-Olivé FX, Kahn K, Tollman S, Norris SA (2014). Barriers to and facilitators of the provision of a youth-friendly health services programme in rural South Africa. BMC Health Serv Res.

[27] Kumar R, Prinja S, Lakshmi PV (2008). Health care seeking behavior of adolescents: comparative study of two service delivery models. Indian J Pediatr.

[28] Koon AD, Goudge J, Norris SA (2014). Considerations for linking South Africa’s youthfriendly services to its community health worker programme. S Afr J CH.

